# Difluoromethylornithine (DFMO), an Inhibitor of Polyamine Biosynthesis, and Antioxidant *N*-Acetylcysteine Potentiate Immune Response in Mice to the Recombinant Hepatitis C Virus NS5B Protein

**DOI:** 10.3390/ijms22136892

**Published:** 2021-06-26

**Authors:** Ekaterina I. Lesnova, Olga V. Masalova, Kristina Yu. Permyakova, Vyacheslav V. Kozlov, Tatyana N. Nikolaeva, Alexander V. Pronin, Vladimir T. Valuev-Elliston, Alexander V. Ivanov, Alla A. Kushch

**Affiliations:** 1 Gamaleya National Research Center of Epidemiology and Microbiology, Ministry of Health of the Russian Federation, 123098 Moscow, Russia; wolf252006@yandex.ru (E.I.L.); kristusha164@mail.ru (K.Y.P.); hyperslava@yandex.ru (V.V.K.); tatyananik.55@mail.ru (T.N.N.); proninalexander@yandex.ru (A.V.P.); vitallku@mail.ru (A.A.K.); 2 Federal State Budgetary Educational Institution of Higher Education “Moscow State Academy of Veterinary Medicine and Biotechnology—MVA by K.I. Skryabin”, 109472 Moscow, Russia; 3 Center for Precision Genome Editing and Genetic Technologies for Biomedicine, Engelhardt Institute of Molecular Biology, Russian Academy of Sciences, 119991 Moscow, Russia; gansfaust@mail.ru

**Keywords:** hepatitis C virus (HCV), nonstructural protein NS5B, *N*-acetylcysteine (NAC), difluoromethylornithine (DFMO), CpG ODN 1826, adjuvants, immune response, HCV vaccine, myeloid-derived suppressor cells (MDSC), regulatory T cells (Treg)

## Abstract

Hepatitis C virus (HCV) is one of the main triggers of chronic liver disease. Despite tremendous progress in the HCV field, there is still no vaccine against this virus. Potential vaccines can be based on its recombinant proteins. To increase the humoral and, especially, cellular immune response to them, more effective adjuvants are needed. Here, we evaluated a panel of compounds as potential adjuvants using the HCV NS5B protein as an immunogen. These compounds included inhibitors of polyamine biosynthesis and urea cycle, the mTOR pathway, antioxidants, and cellular receptors. A pronounced stimulation of cell proliferation and interferon-γ (IFN-γ) secretion in response to concanavalin A was shown for antioxidant *N*-acetylcysteine (NAC), polyamine biosynthesis inhibitor 2-difluoromethylornithine (DFMO), and TLR9 agonist CpG ODN 1826 (CpG). Their usage during the immunization of mice with the recombinant NS5B protein significantly increased antibody titers, enhanced lymphocyte proliferation and IFN-γ production. NAC and CpG decreased relative Treg numbers; CpG increased the number of myeloid-derived suppressor cells (MDSCs), whereas neither NAC nor DFMO affected MDSC counts. NAC and DFMO suppressed NO and interleukin 10 (IL-10) production by splenocytes, while DFMO increased the levels of IL-12. This is the first evidence of immunomodulatory activity of NAC and DFMO during prophylactic immunization against infectious diseases.

## 1. Introduction

Hepatitis C virus (HCV) is one of the leading causes of chronic liver diseases including its end-stage pathologies—cirrhosis and hepatocellular carcinoma. Up to 80% of all acute hepatitis C cases turn into chronic hepatitis C (CHC) [1,2]. As a result of development of direct-acting antivirals (DAAs) and their introduction into clinical practice in early 2010s, CHC has become curable for more than 90% of the patients. However, eradication of the disease is still hampered by several factors. First, such DAAs are not widely accessible for patients, as their price in many countries remains very high. For example, the majority of Russian patients, whose treatment is covered by the government, are treated with interferon-containing regimens, whereas many of those who are willing to take interferon-free schemes face the illegal market of drugs licensed for India and other countries. Second, for some patients, viral RNA is still detected in the liver or blood cells after achievement of sustained virologic response, i.e., after the disappearance of HCV RNA from plasma of peripheral blood [3,4]. In addition, reactivation of other viruses was observed during or after DAA therapy [5,6]. Importantly, DAAs do not reduce risks of virus-associated end-stage liver diseases to the level of the general population [7]. Therefore, attempts to develop prophylactic and therapeutic vaccines against hepatitis C that were paused upon bringing the first DAAs to the market have again been intensified in recent years. The lack of a vaccine can be one of the major obstacles to hepatitis C control [8].

Development of vaccines against hepatitis C is based on several approaches including the search for virus-specific components and compounds that enhance their action. Widely used licensed adjuvants like aluminum hydroxide and water-oil emulsions induce a predominantly humoral response [9,10]. However, the vaccine that can clear the infection should induce not only a humoral response, but also a T cell one. In case of hepatitis C, it has been shown that an effective vaccine should trigger a potent multiepitope and functional Th1 immune response [11]. The spectrum of adjuvants that potentiate Th1 functions is relatively small. At the same time, during HCV infection, T cell response is blocked via several mechanisms including expansion of myeloid-derived suppressor cells (MDSC) and regulatory T cells (Treg) [12,13,14,15]. MDSCs represent a heterogeneous population of immature myeloid cells. Their quantity is relatively low in the normal state; however, the amount of these cells during various pathologies is significantly increased, contributing to the pathogenesis of a disease. MDSCs could exhibit a strong immunosuppressive effect towards T cells via multiple mechanisms such as expression of arginase or inducible NO synthase (iNOS) and overproduction of reactive oxygen species (ROS) [16].

One of the possible approaches to blocking the negative effect of MDSCs on immune response is to influence the toll-like receptor (TLR) signaling that is involved in MDSC regulation [17]. TLR agonists can modulate the suppressive function of MDSCs [18]. These include the derivatives of synthetic oligonucleotides bearing nonmethylated CpG motifs (CpG ODN) that are recognized by TLR9. It has been demonstrated that such compounds can effectively enhance immune response to ovalbumin [19], recombinant viral proteins [20], and DNA [21]. However, in different cell types (in macrophages, dendritic cells, cDCs, pDCs), TLR9 can initiate different signaling pathways [22]. In addition, some sequences of CpG ODN induce transcriptional responses that are strongly biased either towards the expression of inflammatory cytokines or the expression of cytokines and IFN [23].

Pathogens use different strategies to downregulate TLR signaling. For example, the NS3/4A serine protease of HCV cleaves TLR adaptor protein TRIF to inhibit immune response [24]. The regulation of the inflammatory response in peripheral blood mononuclear cells depends on the metabolic status [25,26]. Future studies are required to determine the exact mechanisms of TLR signaling and other molecular pathways induced by the HCV to escape host immunity.

Many other classes of compounds can regulate immune response via various cell signaling and metabolic pathways. In this respect, much attention is now given to metabolic and redox-sensitive pathways, as immune cell differentiation and proliferation requires reshaping of their metabolic profiles and is achieved through redox-sensitive transcription factors including NF-κB, STAT3, and others. For example, the mTOR pathway has an essential role in generating and regulating immune cells to combat pathogens, including the generation of CD8+ T cell effector and memory populations; these functions could be modulated in order to enhance vaccine efficacy and antitumor immunity [27]. The mechanistic target of rapamycin plays an important immunoregulatory role in the germinal center in generating high-affinity antibodies [28]. Therefore, to analyze the possible immunostimulatory activity of the mTOR pathway, the inhibitor of mTORC1 complex formation, rapamycin, should be evaluated since it can control immune responses [29].

Biogenic polyamines play relevant immunostimulatory roles in immune cell differentiation, activation, and recruitment [30]. Ornithine decarboxylase (ODC), the rate-limiting enzyme in polyamine biosynthesis, synthesizes putrescine that has been shown to impair polarization of naïve macrophages to the classical proinflammatory M1 response [31]. Inhibitors of polyamine-metabolizing enzymes are also implicated in antitumor immunity. ODC inhibitor α-difluoromethylornithine (DFMO) enhances antitumor CD8+ T cell infiltration and IFN-γ production as well as impairs the MDSCs’ suppressive activity [32]. Similar data were obtained by E.T. Alexander et al. in 2017 [33]: blocking polyamine biosynthesis with DFMO significantly inhibited tumor growth via an increase in granzyme B+, IFN-γ+ CD8+ T cells and a decrease in immunosuppressive tumor-infiltrating cells including MDSCs and Tregs.

Antioxidants are involved in scavenging reactive oxygen species (ROS) and reactive nitrogen species (RNS), thereby preventing oxidation of lipids in membranes, DNA, and proteins and also affecting redox-sensitive signaling pathways. Therefore, in human clinical trials, *N*-acetylcysteine (NAC), one of the best known antioxidants, has been shown to replenish glutathione stores and increase the proliferative response of T cells. NAC has also been attributed much attention in connection with respiratory viral infections [34], SARS-CoV-2 in particular. A decrease in the viral load can occur through NAC’s interference with cellular redox status via serving as a precursor for glutathione biosynthesis, thus alleviating the detrimental effect of virus-induced oxidative stress and concomitant cell death, as well as by improving T cell response and modulating inflammation [35]. NAC prevents apoptosis induced by proapoptosis Fas antigen/Fas ligand lethal signal that is upregulated during virus infections, such as HIV, HCV, and influenza [36]. This antioxidant also markedly reduces levels of TNF-α and decreases levels of oxidative stress markers in patients with pneumonia [37].

In this study, we investigated the possibility of using compounds that act on the cellular receptors and molecular pathways that affect the metabolism and functional activity of immune cells. As an immunogen, we used the HCV NS5B protein that is an RNA-dependent RNA polymerase—the critical component of the virus replicase. Our goal was to analyze the immunostimulatory activity of inhibitors of polyamine biosynthesis, the mTOR pathway, redox systems regulators, and cellular receptors during immunization of mice with the NS5B protein.

## 2. Results

### 2.1. Induction of MDSCs and Tregs Correlates with Suppression of the Cellular Immune Response

**Figure 1:**
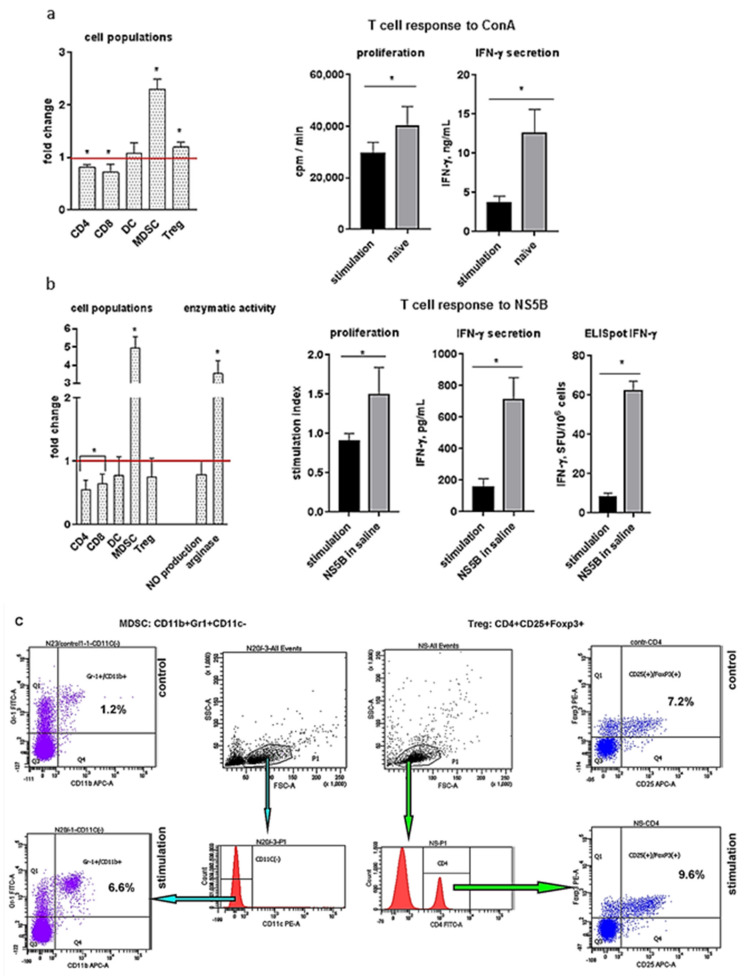
An increase in the populations of suppressor cells in the spleen of mice causes a decrease of the cellular immune response. To increase the level of suppressive cells in spleens, the mice (*n* = 3) were injected with CpG ODN 1826 + IFA SC, and after 10 days, the T cell response of lymphocytes to ConA was evaluated (**a**). The mice (*n* = 3) were IP injected with an IFA (0.5 mL/mouse, six times weekly) accompanied by triple SC immunization with the NS5B protein (4 µg/mouse), and after 9 days, we evaluated the T cell response of lymphocytes to NS5B (**b**). The values of cell populations and enzymatic activity obtained were normalized to the values obtained for the control mice (red lines); * *p* < 0.05 compared to the group of naïve mice (**a**) and the group of mice immunized with NS5B in saline (**b**). (**c**) Gating strategies and representative dot plots for MDSCs (left) and Tregs (right). The splenocytes were stained with anti-CD4, anti-CD8, anti-CD11c, anti-CD11b, anti-Gr1, anti-CD25, and anti-Foxp3 antibodies and analyzed by flow cytometry. To determine MDSCs, a population of lymphocytes was isolated (gate P1), and cells from this gate were used to identify CD11c(−) cells. The CD11c(−) dot plot was used to determine the proportion of Gr-1+, CD11b+ cells. To determine Tregs, in the lymphocyte gate (P1), a population of CD4+ T cells was isolated. The CD4+ dot plot was used to determine the proportion of CD25+, FoxP3+ T cells.

To evaluate the effect of suppressor cells on T cell immune response, two models of MDSC and Treg stimulation were used. First, analysis of splenocyte receptors after administration of emulsion CpG ODN 1826 in an IFA revealed that the relative number of CD4+ and CD8+ T cells decreased by 1.2–1.4-fold (*p* < 0.05). In contrast, relative levels of suppressor cells were significantly increased after stimulation. The most pronounced effect was observed for MDSCs, the level of which increased by more than twofold (Figure 1a). In vitro evaluation of T cell response to ConA showed that in a group of stimulated suppressor cells, proliferation decreased by 1.7-fold, IFN-γ production—by 3.4-fold (*p* < 0.05).

In the second model of MDSC induction, an IFA was administered six times. It demonstrated similar effects: a decrease of relative levels of CD4+, CD8+ cells and an increase of the MDSC population by more than fivefold (Figure 1b). Accumulation of MDSCs was accompanied by increased levels of arginase activity in suppressor cells and reduced T cell response to NS5B.

These data suggest that an increase in the number of MSCSs and, to a lesser extent, of Tregs contributes to suppression of the T cell function in vivo.

### 2.2. Analysis of the Action of Compounds on T Cell Functional Activity

**Figure 2:**
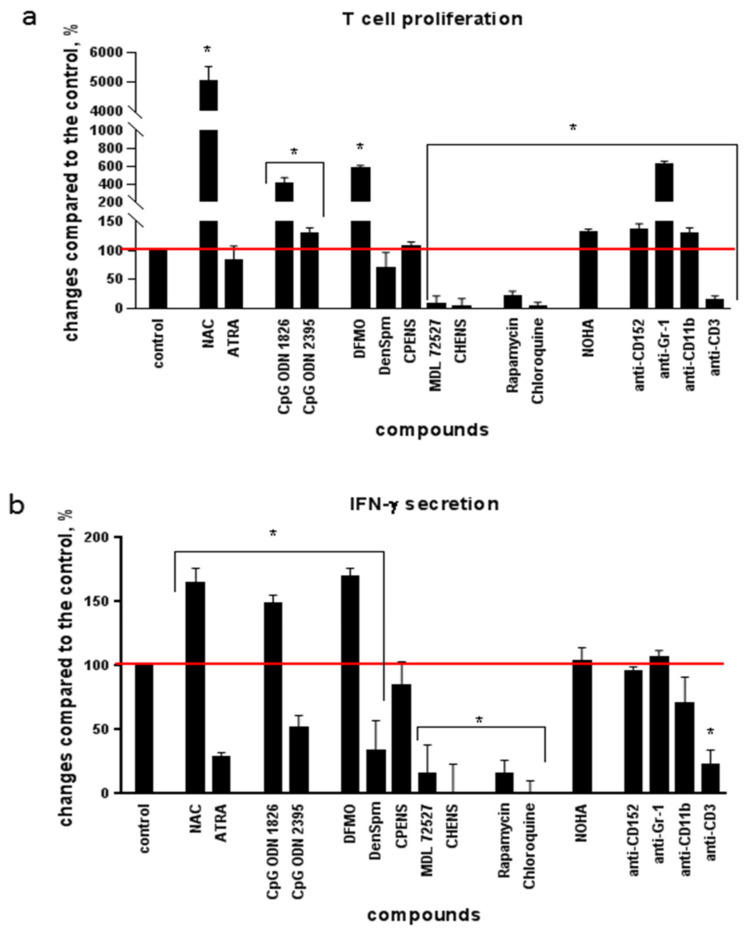
The effect of the studied compounds on the proliferative activity of splenocytes (**a**) and the secretion of IFN-γ (**b**) in response to concanavalin A. The lymphocytes were seeded onto 96-cell plates and incubated with the compounds and ConA for 3 days at 37 °C in a humid atmosphere containing 5% CO_2_; then, lymphocyte proliferation and IFN-γ secretion assays were performed. The values obtained in the samples without compounds (red lines) are taken as 100%; * *p* < 0.05 compared to the control (samples with media alone).

Next, the splenocytes obtained from the mice with a notable increase in the MDSC population and suppressed T cell response were treated ex vivo with the compounds that could affect T cell functions. The latter were evaluated using two parameters, namely lymphocyte proliferation and secretion of IFN-γ in response to ConA. As the positive control, the untreated samples were used, whereas similar cells treated with the antibodies to murine CD3 receptors were used as the negative control. These antibodies almost completely blocked the activity of T cells in response to ConA through binding to their receptors (Figure 2a,b). Evaluation of 16 compounds (Table 1, Figure 2) revealed that downregulation of the activity of arginase, an enzyme that is produced by MDSCs, with its specific inhibitor NOHA led to a 30% increase in T cell proliferation (Figure 2a). A similar effect was observed in case of blocking of Treg functions with antibodies to CD152. A more pronounced stimulation of lymphocyte proliferation was detected for antibodies to granulocyte Gr-1 receptor and for three low-molecular-weight compounds: antioxidant *N*-acetylcysteine (NAC), TLR9 ligand CpG ODN 1826, and polyamine biosynthesis inhibitor DFMO. However, other compounds studied either suppressed the T cell response or did not affect it. Hence, this experiment allowed identifying three compounds that had the highest stimulatory effect on in vitro proliferation and IFN-γ secretion: NAC, DFMO, and CpG ODN 1826. Noteworthily, the repeated experiments with different concentrations of these three compounds showed that their effect on the immune response in vitro was dose-dependent. For NAC, the effective concentrations were 5–10 mM, for DFMO—10 mM, for CpG ODN 1826—1.5–3.5 µM.

### 2.3. NAC and DFMO Potentiate Immune Response of Mice to the HCV NS5B Protein

**Figure 3:**
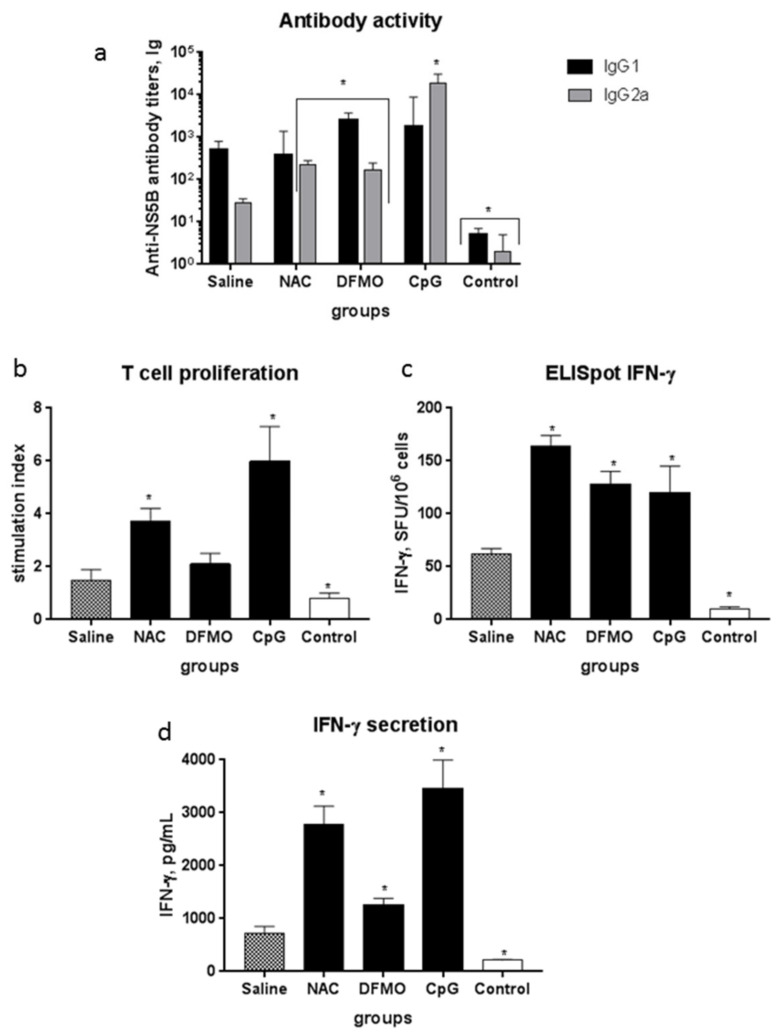
Increasing the immune response of mice to the recombinant protein HCV NS5B using selected compounds. Each group (five mice) received the recombinant protein NS5B at a dose of 4 µg/mouse in three injections with two-week intervals. NS5B was injected subcutaneously in combination with saline or CpG, whereas NAC and DFMO—with drinking water. (**a**) The levels of anti-NS5B antibodies in the sera of mice are expressed as endpoint titers in ELISA. To assess the cellular response of lymphocytes in vitro, we used purified recombinant proteins HCV NS5B; medium alone was used as the negative control. (**b**) The results of T cell proliferation are expressed as stimulation indexes (SI); the IFN-γ production by splenocytes in response to NS5B was assayed as the number of IFN-γ-synthesizing cells by ELISpot in the number of spot-forming cells (SFC) per 10^6^ cells (**c**) or as cytokine concentrations in culture fluids by ELISA, expressed as pg/mL (**d**) (* *p* < 0.05 compared to the group without an adjuvant (saline)).

The next step was to evaluate these compounds as enhancers of the immune response to HCV in vivo. Analysis of the response in the first series of experiments, during which the recombinant NS5B protein was injected IP in a combination with NAC or DFMO demonstrated no enhancement of humoral or T cell response compared to immunization with NS5B in the absence of these compounds (Table S1). The absence of adjuvant effect could result from rapid clearance of both compounds from the organisms [38,39]. Therefore, the scheme of their administration was modified: in the second set of experiments, both compounds were added to drinking water. The results of evaluation of humoral and T cell response in the mice are presented on Figure 3.

Humoral response was evaluated using indirect ELISA with quantification of IgG1 and IgG2a titers to NS5B. In the mice that received the NS5B protein without potential adjuvants, the titers of the IgG1 isotype of antibodies were increased using DFMO and CpG by fivefold and 3.5-fold, respectively (Figure 3a). The levels of IgG2a were increased in the case of all the three compounds: NAC and DFMO elevated their levels by 4–5-fold, whereas in the case of CpG, the increase was remarkable—it reached 670-fold. Immunization of mice with NS5B alone did not affect lymphocyte proliferation in response to the protein, while NAC and CpG increased the proliferative activity of the splenocytes from the immunized mice (Figure 3b) (*p* < 0.05). In the case of DMFO, a moderate increase in proliferation was also noted; however, it did not reach the cutoff of statistical significance (*p* = 0.053). Finally, all these three compounds significantly stimulated IFN-γ synthesis (by 2–2.5-fold) and secretion in response to NS5B (by 4–5-fold) (Figure 3c,d).

**Figure 4:**
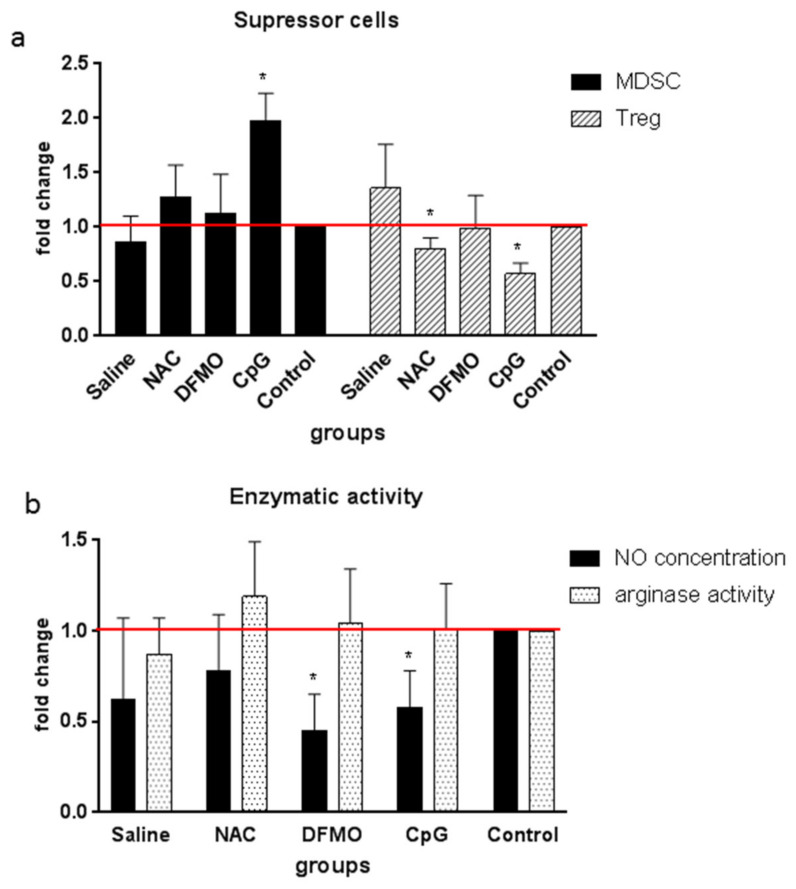
Changes in the content of suppressor cells (**a**) and enzymatic activity (**b**) of splenocytes of the mice immunized with recombinant protein HCV NS5B using candidate adjuvants. Each group (five mice) received the recombinant protein NS5B at the dose of 4 µg/mouse in three injections with two-week intervals. NS5B was injected subcutaneously in combination with saline or CpG, whereas NAC and DFMO—with drinking water. The values obtained were normalized to the values obtained for the mice of the control group (red lines); * *p* < 0.05 compared to the control group.

Another goal was to reveal the association between adjuvant properties of the compounds with changes in the immune cell proportion and some of their functions. Investigation of spleen cells from the immunized mice showed that in the case of CpG, the relative population of MDSCs among splenocytes increased by almost twofold, whereas the population of Tregs decreased both for this compound and for NAC (Figure 4a). The relative numbers of CD4+, CD8+ lymphocytes as well as of dendritic CD11c+ cells did not change (Table S2). Nitric oxide production in the DFMO and CpG groups decreased, while the activity of arginase was unaffected (Figure 4b).

### 2.4. Immunoregulatory Properties of the Compounds

**Figure 5:**
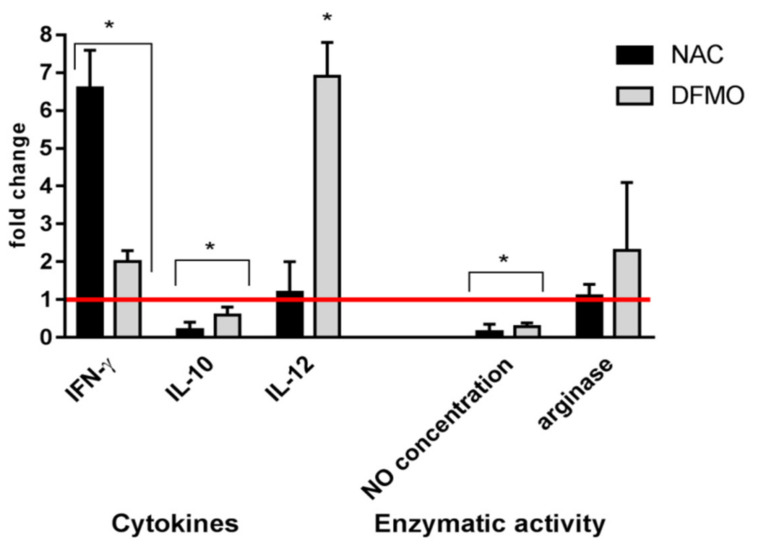
Changes in cytokine secretion and enzymatic activity of the splenocytes treated with NAC and DFMO. Lymphocytes of the intact mice were incubated with the compounds for 3 days at 37 °C in a humid atmosphere containing 5% CO_2_. Cytokines were quantified by ELISA in conditioned media, and enzymatic activity was measured in cell lysates. The values obtained were normalized to the values obtained for the samples without compounds (red line). The values in each diagram are the means ± SD of four measurements done in three independent experiments; * *p* < 0.05 compared to the control (samples with media alone).

To clarify the mechanisms of the compounds’ action, the splenocytes isolated from a new group of intact mice were treated with NAC or DFMO and evaluated ex vivo for the expression of arginase, production of nitric oxide, and secretion of cytokines (Figure 5). It was demonstrated that neither NAC nor DFMO affected arginase activity but decreased levels of NO production—by seven- and fourfold, respectively. Both compounds also potentiated the production of IFN-γ and decreased the production of IL-10. DFMO caused a sevenfold enhancement of IL-12.

**Figure 6:**
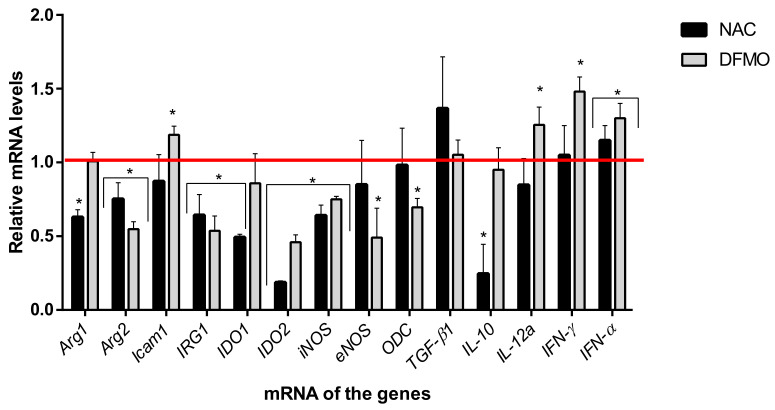
Changes in the transcriptional activity of genes under the influence of NAC and DFMO. Lymphocytes of the intact mice were incubated with the compounds for 3 days at 37 °C in a humid atmosphere containing 5% CO_2_. Quantitative analysis of gene expression in cell lysates was performed by real-time PCR by comparing the threshold number of cycles for the target genes and the reference GUS RNA. The values obtained were normalized to the values obtained for the samples without compounds (red line). The values in each diagram are the means ± SD of two measurements done in three independent experiments; * *p* < 0.05 compared to the control (samples with media alone).

Next, analysis of transcription of 14 genes involved in the regulation of immune response was performed. For 13 of them, statistically significant changes were found (Figure 6). Noteworthily, the compounds significantly downregulated transcription genes of arginase 2, IRG, IDO2, and iNOS compared to the control group. In addition, NAC reduced the mRNA levels of arginase 1, IDO1, and DFMO—an eNOS and ODC, but upregulated ICAM-1. For cytokine genes, an increase in the expression of type I interferon, IFN-α, was found, whereas IFN-γ and IL-12 were upregulated only during incubation with DFMO, and IL10 was downregulated by NAC.

## 3. Discussion

Vaccination is one of the most effective ways of combatting infectious diseases since vaccines can prevent virus spread. However, despite a tremendous progress in the development of prophylactic vaccines against poliomyelitis, tetanus, smallpox, in case of many other widespread and dangerous diseases including hepatitis C, there are still no effective vaccines.

Development of effective and safe vaccines against hepatitis C requires a search for novel adjuvants with high protective activity lacking the drawback of the currently used analogs. In this study, we investigated the immunostimulatory action of a series of biologically active compounds including two synthetic oligodeoxynucleotides bearing unmethylated CpG motifs (CpG ODN). Many groups have reported that the compounds of this class activate mechanisms of immune response to various pathogens [40]. They are known to act as adjuvants during immunization by viral antigens [41,42], including the HCV E2 glycoprotein [43]. Therefore, this type of adjuvants was chosen in our study for the comparison of adjuvant properties of other, less studied compounds. Additional rationale for such a comparison was provided by certain drawbacks of CpG ODN such as instability, inhibition of Th2-adaptive immunity, and, in some cases, even immunosuppressive action during systemic application/usage [44,45,46]. Furthermore, unfavorable pharmacokinetics/biodistribution patterns, high binding to plasma proteins, and lack of specificity for target cells have been noted [47,48,49]. Thus, the search for an optimal adjuvant as a critical component of an anti-HCV vaccine remains an important task.

Our choice of compounds was based on observations from various groups that inflammation is accompanied by the spread of MDSCs in the organism, in the liver in particular [50]. The data on MDSCs during chronic hepatitis C are contradictory. Some groups did not observe any increase in MDSC levels in the peripheral blood of CHC patients [51], whereas the others demonstrated that HCV upregulates the number of MDSCs [13,14,15]. Therefore, we used two approaches to assess the MDSCs’ effect on immune response to the recombinant NS5B protein that allowed imitating activation of these cells during hepatitis C in vivo experiments. In both cases, an incomplete Freund’s adjuvant was used, which interacted with NOD2 [52,53]. HCV virions or the HCV core protein induced MDSC-suppressive monocytes via TLR2 [14]. TLR signaling could induce MDSC accumulation and enhance the ability to inhibit T cell responses due to the activation of MyD88-dependent signaling pathways that activate NF-κB, IL-6, TNF-α, iNOS, and Arg-1 expression as well as enhanced ROS production [23]. As a result, we observed downregulation of the T cell response, i.e., by proliferation, synthesis, and secretion of IFN-γ in response to mitogen ConA or to the NS5B protein.

The goal was to block suppressive action of MDSCs and to potentiate immune response to the viral protein. It has been accounted that MDSC’s action can be achieved through direct cell contacts via cellular receptors or soluble mediators [16]. MDSC regulation by the HCV involves the TLR signaling and the intracellular molecular pathways, including PI3K/AKT/mTOR, that are crucial for cell viability during stress conditions [13,14]. Therefore, the compounds listed in Table 1 were chosen to regulate spleen cells ex vivo. They included the molecules that targeted cellular receptors of immunocytes (Nos. 3, 4, and 13–16), cellular mediators (Nos. 1, 2, 11, and 12), and molecular metabolic pathways (biogenic polyamines—Nos. 5–9; and mTOR—No. 10). T cell response analysis revealed that the most pronounced stimulation of immune response was triggered by three compounds: NAC, DFMO, and CpG ODN 1826 (one of the two oligonucleotides studied differing in backbone composition and the CpG motif). Note that immune response was stimulated by treating splenocytes ex vivo with the same CpG ODN that was used to activate suppressor cells in vivo. The differences in the direction of action, apparently, could be explained by the introduction in vivo of not only the TLR9 agonist (CpG ODN), but also of the NOD2 agonist (IFA) or, in the second experiment, an additional viral protein acting on the TLR surface receptors. It has been shown that TLR agonists can both activate the immunosuppressive activity of MDSCs and block it [17]. Molecular mechanisms of regulating MDSCs by TLR are still relatively limited. It can be assumed that the activation of the T cell response observed under the action of CpG ODN occurs when the signaling pathway is switched with the adapters TRIF and MyD88 and downstream signaling proteins.

Another finding of our study was that NAC, DFMO, and CpG ODN 1826 displayed a different action on immune response in the mice that received the NS5B protein, one of the key components of candidate vaccines against hepatitis C. All the three compounds enhanced the specific activity of IgG2a antibodies to NS5B, while DFMO enhanced the specific activity of both IgG1 and IgG2a antibodies. It is generally accepted that IgG1 antibodies reflect Th2 response and synthesis of proinflammatory cytokines (IL-4, IL-10, IL-13, and IL-17). In contrast, the IgG2a subtype is a marker of Th1 response and synthesis of another set of proinflammatory cytokines (IFNs, IL-1β, IL-6, IL-12, and TNF-α). Special attention should be paid to IFN-γ production since this cytokine exhibits immunomodulatory and antitumor activity and is crucial for protection against viral infections. All the three evaluated adjuvants significantly increased the number of IFN-γ-producing cells and secretion of this cytokine in response to immunization with the NS5B protein. The addition of NAC to the immunization regimen reduced the number of Tregs and suppressed the synthesis of NO and the production of IL-10 in the spleen. CpG ODN decreased the number of Tregs and NO concentrations. Neither NAC nor DFMO affected the levels of MDSCs, whereas the usage of CpG ODN during immunization led to their twofold increase compared to the control. Interestingly, an increase in the amount of MDSCs during immunization with a combination of NS5B and CpG ODN did not suppress the immune response but augmented it, unlike in a natural HCV infection. One can assume that the mechanisms of MDSC regulation in these two situations may differ. This is supported by our data showing that immunization of mice with the NS5B protein did not affect arginase expression and activity, whereas some data clearly show that the increase in MDSC levels is associated with T cell suppression via the induction of arginase that catabolizes amino acid arginine [16]. It should be noted that a decrease in Arg2 expression in the case of DFMO and NAC led not to the induction of iNOS that also utilizes arginine but to its downregulation. One cannot exclude the possibility that the compounds studied can stimulate differentiation of MDSCs into mature myeloid cells that do not exhibit immunosuppressive functions, similarly to what was previously shown for all-trans retinoic acid (ATRA) [54]. The possibility of such an action is indicated as an increase in the expression of mRNA of IFN-α upon incubation with DFMO and NAC since IFN-α is the key effector for the induction of MDSC maturation [18]. The authors showed that the treatment of mice with recombinant IFN-α is sufficient to block MDSC-suppressive action.

Our study showed that DFMO and NAC increased the level of IFN-γ both in vitro and in vivo; this cytokine is the key player of the antiviral defense system. Quantification of mRNA levels showed for the first time that DFMO upregulates the expression of adhesion molecule ICAM1 which is a master regulator of cellular responses in inflammation and injury resolution [55]. At the same time, the selected compounds downregulated transcription of the major suppressive function mediators including redox system components (NO, NOS) and metabolic pathways such as urea cycle (arginase), tryptophan catabolism (indolamine-2,3-dioxygenases IDO1 and IDO2), and polyamine biosynthesis ornithine decarboxylase (ODC). Both compounds downregulated the transcriptional level of mitochondrial enzyme immune-responsive gene 1 (IRG1), which produces itaconate under inflammatory conditions, principally in cells of myeloid lineage. Itaconate exerts anti-inflammatory effects in vitro and in vivo and limits production of IL-1β, IL-18, IL-6, IL-12, but not of TNF-α [56]. It is worth noting that all these pathways are interconnected: (i) polyamines are synthesized from non-proteinogenic amino acid ornithine—a product of arginase; (ii) NO is produced by NO synthases that catalyze the direct conversion of arginine into citrulline, another metabolite of the urea cycle; (iii) both polyamine pathways and tryptophan catabolism processes are associated with ROS production, albeit in different forms: H_2_O_2_ in the case of polyamines and singlet oxygen in the case of tryptophan [57].

One can see that several parameters of adjuvant activity are observed both at mRNA and protein levels and in the immune cell phenotype/quantity. This demonstrates that enhancement of immune response in response to the HCV protein is achieved via various cellular and molecular mechanisms.

NAC is a precursor of glutathione, one of the most abundant antioxidants and a cofactor of many antioxidant enzymes. Although it cannot scavenge ROS directly, it counteracts the production of ROS and reactive nitrogen species (RNS). NAC has been shown to enhance proliferation of T cell clones, increase live, and protect T cells from hazardous effects of ROS [58]. It is widely used in clinical practice and as mucolytic agents; moreover, recent data clearly show that this thiol protects cells by reducing the cytopathogenic effect of many diseases including viral infections [59].

DFMO is an irreversible inhibitor of ornithine decarboxylase—an enzyme that catalyzes the rate-limiting step of polyamine biosynthesis. Polyamines such as spermine, spermidine, and their analogs are ubiquitous low-molecular-weight oligocations present in all types of prokaryotic and eukaryotic cells at millimolar and submillimolar concentrations. Polyamines seem to be indispensable for efficient replication of DNA and RNA viruses as the inhibitors of their biosynthesis often exhibit antiviral activity [60,61,62]. It has been shown for Zika and Chikungunya viruses, with the most pronounced effect seen upon pretreatment of cells with the compounds [61]. Similar data also exist for hepatitis C virus. Moreover, we previously showed that HCV does alter polyamine biosynthesis [62]. ODC is also linked to inflammation: during an inflammatory condition in intestines, macrophages exhibit lower activity but elevated levels of ODC expression [63]. In a colitis model, the authors demonstrated that the loss of the ODC gene leads to pronounced induction of inflammatory cytokines including GM-CSF, TNF-α, IL-1α and IL-1β, and IFN-γ as well increased macrophage polarization towards M1. The mice lacking the *ODC* gene in myeloid cells exhibited an improved intestine structure and decreased levels of inflammation and epithelium damage. It should be noted that DFMO (eflornithine) is an FDA-approved drug that features high safety and tolerability.

To sum up, our study is the first evidence that NAC and DFMO can be used as compounds that enhance the immune response—the adjuvants with immunomodulatory properties, as demonstrated in experiments on the immunization of mice with the recombinant NS5B protein. Both compounds should be used permanently during animal immunization, i.e., added to drinking water. It is tempting to speculate that a combination of DFMO and NAC can exhibit a synergistic effect during immunization with viral antigens since immune and cellular mechanisms of these compounds are different. One cannot exclude the possibility that such a combination can be advantageous compared to CpG ODN due to both decreased influence of MDSCs and IDOs that are often elevated during CpG ODN usage [45] and enhanced IFN-γ production. These results show that an investigation of new adjuvants can contribute to the field of vaccine development against HCV as well as against other viruses.

## 4. Materials and Methods

### 4.1. The Immunogen

The recombinant NS5B protein lacking hydrophobic 21 amino acid residues on the C-terminus of the polypeptide chain was used as a virus-specific component for mouse immunization and stimulation of T cells in vitro, as well as an antigen for the ELISA assay for antibody determination. This protein, corresponding to 2420–2990 amino acid residues of the HCV polypeptide (genotype 1b), was expressed in *Escherichia coli* and purified on Ni-NTA agarose as described previously [64].

### 4.2. The Compounds

The following compounds were used as potential blockers of the suppressor cells and immunostimulatory agents (Table 1). The nontoxic concentrations were selected based on the analysis of the literature data and the preliminary experiments using the MTT assay.

### 4.3. Mice

Mice of the DBA/2J (H-2d) line (females, 6–8-week-old) were obtained from the laboratory of animal breeder Stolbovaya, FMBA, Moscow Region. All the ex vivo and in vivo animal experiments were carried out in accordance with order 199n of the Ministry of Health of the Russian Federation and with the “Regulations on the Ethical Attitude to Laboratory Animals of N.F. Gamaleya NRCEM (Moscow, Russia)”.

### 4.4. Lymphocyte Proliferation Assay

Analysis of lymphocyte proliferation during treatment of the compounds was carried out as described previously [65]. Splenocytes were isolated from the mice, washed with the RPMI-1640 medium (Paneco, Moscow, Russia), and seeded onto 96-well plates at a density of 5 × 10^6^ per well in the RPMI-1640 medium supplemented with 20% fetal calf serum (Invitrogen, Waltham, MA, USA), 2 mM glutamine, 4.5 mg/mL glucose, 50 µg/mL gentamycin, and 0.2 U/mL insulin. The compounds (Table 1) were added at the concentrations chosen from the preliminary experiments. Concanavalin A (ConA, Paneco) was used at the final concentration of 5 µg/mL. For the negative control, the medium alone was used. After 72-h incubation at 37 °C in a humid atmosphere with 5% CO_2_, the conditioned medium was removed for the quantification of cytokines. A fresh medium containing [^3^H]-thymidine (1 µCi/well) (TdR, Amersham-Pharmacia-Biotech, Amersham, UK) was added, and 18 h later, the cells were harvested onto glass fiber filters. Radioactivity was measured using a MicroBeta2 β-counter (PerkinElmer, Waltham, MA, USA). The proliferation stimulation index (SI) was calculated as the ratio of radioactivity (cpm/min) in the wells with the compound to the level of radioactivity in the control wells.

### 4.5. Mouse Immunization

Two series of experiments were conducted to test the immunostimulatory activity of the compounds upon immunization of mice with the recombinant HCV NS5B protein. Each group (five mice) received the protein at the dose of 4 µg/mouse in three injections with two-week intervals. Humoral and cellular immunity was examined on days 7–9 after the third immunization. In the first series of experiments, NS5B was injected intraperitoneally (IP) in saline or in combination with NAC and DFMO (Table 2). The doses of both compounds were selected based on the literature data from the study of experimental tumor and oxidative stress in mice [66,67].

In the second set of experiments, the mice, distributed between four groups, received NS5B by subcutaneous injections (SC) without adjuvants or in combination with CpG, NAC and DFMO—with drinking water (Table 2). We controlled consumption of drinking water in the mice, which turned out to be about 3 mL per day. NAC concentration in water was 40 mM, equivalent to 1 g/kg body weight/day [68]. DFMO concentration in drinking water was 0.2%, equivalent to 0.3 g/kg body weight/day—a safe dose during long-lasting experiments in animals [69]. The NAC and DFMO solutions were stored in dark bottles and replaced with fresh ones twice a week. The control group received a 0.9% NaCl solution instead of the NS5B protein.

### 4.6. Increasing the Level of Suppressive Cells in the Spleen

We took into account the data that systemic administration of CpG ODN in an incomplete Freund’s adjuvant (IFA) induced expansion of splenic myeloid CD11b+ Gr1+ cells and that these cells caused suppression of T cell proliferation [70,71]. We injected a CpG ODN 1826+ IFA subcutaneously, and after 10 days, we evaluated the T cell response of lymphocytes to ConA. The second approach consisted in regular IP injections of an IFA (0.5 mL/mouse, six times weekly) accompanied by triple SC immunization with the NS5B protein (4 µg/mouse) with a similar evaluation of cellular response as described below.

### 4.7. Humoral Immune Response

The levels of interaction of antibodies in mouse serum samples with the NS5B protein were quantified by indirect ELISA as described previously [72] using antibodies against mouse Ig isotypes IgG1 and IgG2a conjugated with horseradish peroxidase (Jackson Immunoresearch Laboratories, West Grove, PA, USA).

### 4.8. T Cell Proliferation and ELISpot Assays

Proliferation of lymphocytes of the immunized mice was assessed in vitro by quantification of incorporation of [^3^H]-thymidine into lymphocytes. The reaction was carried out as described above; the NS5B protein was added as a specific stimulator to the final concentration of 1 µg/mL. Quantification of the cells secreting IFN-γ was carried out using an ELISpot mouse IFN-γ Kit (BD Bioscience», Franklin Lakes, NJ, USA) in accordance with the manufacturer’s instructions. The stained spots were visualized using an MBS-10 stereomicroscope (Lomo, St. Petersburg, Russia). The results were expressed as the difference in the number of spots (spot-forming units, SFU) per 10^6^ cells between the wells stimulated by the NS5B and the control wells without stimulation (medium alone).

### 4.9. Cytokine Quantification

IFN-γ, IL-10, and IL-12 were quantified by ELISA in the conditioned medium samples obtained in the lymphocyte proliferation assays. The following ELISA systems were used: Mouse IFN-γ ELISA development kit (HRP) (Mabtech, Nacka Strand Sweden), Mouse IL-10 DuoSet ELISA, and Mouse IL-12 p70 Duoset ELISA (R&D Systems, Minneapolis, MN, USA). The sensitivity was 2 pg/mL for IFN-γ and 30 pg/mL for IL-10 and IL-12. The concentrations of cytokines were calculated from the respective calibration curves obtained using the standards from these systems.

### 4.10. Flow Cytometry

The phenotype of cells from the spleen was determined using multicolor flow cytometry. Fluorescently labeled antibodies to clusters of differentiation (CD): phycoerythrin (PE)-labeled antibodies against CD11c or CD8, fluorescein isothiocyanate (FITC)-labeled antibodies against CD4 or Gr-1 (Ly-6G and Ly-6C), allophycocyanin (APC)-labeled antibodies against CD11b or CD25, and PE-labeled antibodies against the intracellular FoxP3 transcription factor (BD Biosciences) were used. Antibodies for the corresponding isotype controls were included in all the experiments. Cell staining was performed according to the standard protocol provided by the manufacturer. In each case, 10^6^ cells per probe were used. For staining the intracellular protein, a kit for cell staining and permeabilization was used (BD Biosciences, USA). Measurements of the absolute and relative number of cells carrying the markers were performed on a BD FACSCanto™ II cytometer (USA), and the results were analyzed using the BD FACSDiva v.6.1.3 software (BD Biosciences, USA).

### 4.11. Arginase Activity Assay

The measurement of arginase activity in lysates of lymphocytes was performed using a QuantiChrom^TM^Arginase Assay Kit (BioAssay Systems, Hayward, CA, USA) which detects urea, the product of the enzymatic reaction. Arginase activity levels were normalized to the total protein content quantified with the Bradford reagent (Sigma, St. Louis, MO, USA) and expressed as U/mL/mg protein.

### 4.12. Nitric Oxide Concentrations

Concentrations of nitric oxide in cell lysates were quantified using a Griess Reagent Kit for Nitrite Determination (Molecular Probes, Eugene, OR, USA) according to the manufacturer’s specifications. The calculated concentration of NO was expressed as µM/mg protein.

### 4.13. Quantification of mRNA Levels

The levels of mRNAs were quantified using reverse transcription and real-time PCR. The lymphocytes were seeded onto six-well plates at a density of 6 × 10^6^/mL and incubated with the compounds for 3 days at 37 °C in a humid atmosphere containing 5% CO_2_. Total RNA was extracted using the ExtractRNA reagent («Evrogen», Moscow, Russia) according to the company’s instruction. The RNA precipitate was dissolved in 30 µL water and treated with DNAse I (1 U/µg RNA) (Roche Holding, Basel, Switzerland) for 15 min at 37 °C with subsequent enzyme inactivation at 75 °C for 10 min. Reverse transcription was performed with the RevertAid H Minus Reverse Transcriptase (Thermo Fisher Scientific Inc., Waltham, MA, USA). Specifically, 1 µg RNA was incubated with 200 U reverse transcriptase at 42 °C for 2 h followed by inactivation for 15 min at 70 °C. Real-time PCR was performed using the primers listed in Table 3 according to the previously described procedure. The levels of mRNA were normalized to the levels of glucuronidase-β (GUS) mRNA and expressed as the fold change compared to the control. The levels of mRNA were quantified in three independent samples with two technical duplicates for each sample.

### 4.14. Statistical Analysis

Statistical analysis was performed using the Statistica 8 and GraphPad Prism 7 software. The data were presented as the means ± SD (standard deviation) from three independent experiments if not stated otherwise. Significant differences between the groups were identified using two-sample Student’s *t*-test or Mann–Whitney test as a commonly used nonparametric alternative to the *t*-test when appropriate. The differences were considered statistically significant at *p* < 0.05.

## Figures and Tables

**Table 1:** Potential stimulators of the immune response used in this study.

No.	Compound	Abbreviation	Supplier	Concentration ^3^	Target, Functions
1	*N*-acetylcysteine	NAC	Sigma	10 mM	Antioxidant
2	All-trans retinoic acid	ATRA	Sigma	0.5 µM	Vitamin A metabolite; exerts potent effects on cell growth, differentiation, and apoptosis
3	Synthetic oligonucleotides bearing unmethylated CpG motifs	CpG ODN 1826, CpG	Invivogen	2.5 µM	TLR9 agonist, class B
4		CpG ODN 2395	Invivogen	2.5 µM	TLR9 agonist, class C
5	2-D,L-difluoromethylornithine	DFMO	^1^	10 mM	Irreversible inhibitor of ornithine decarboxylase (ODC)
6	N1,N11-diethylnorspermine	DenSpm	^2^	20 µM	Inducer of spermine/spermidine N1-acetyltransferase (SSAT)
7	N,N1-bis(2,3-butadienyl)-1,4-diaminobutane	MDL 72527, MDL	Sigma	20 µM	Inhibitor of polyamine oxidases
8	N1-cyclopropylmethyl-N11-ethylnorspermine	CPENS	^2^	10 µM	SSAT inducer
9	N1-cycloheptylmethyl-N11-ethylnorspermine	CHENS	^2^	10 µM	SSAT inducer that does not affect polyamine levels
10	Rapamycin	Rapamycin	Cell Signaling Technology	250 nM	Specific mTOR inhibitor (mTORC1)
11	Chloroquine	Chloroquine	Sigma	20 µM	Inhibitor of autophagy, exhibits the anti-inflammatory effect
12	N^ω^-hydroxy-nor-arginine	NOHA	Sigma	0.1 mM	Arginase inhibitor
13	Antibodies to Tregs	anti-CD152	BD Biosciences	1 µg/mL	CTLA4 antigen expressed in Tregs
14	Antibodies to granulocytes Ly-6G and Ly-6C (Gr-1)	anti-Gr-1	BD	1 µg/mL	Gr-1 antigen expressed on monocytes, neutrophils, and granulocytes
15	Antibodies to CD11b	anti-CD11b	BD	1 µg/mL	Antigen expressed on monocytes, macrophages, NK cells, and granulocytes
16	Antibodies to CD3e	anti-CD3	BD	1 µg/mL	Antigen expressed on T cells

Note: The compounds were a kind gift of ^1^ Prof. Patrik Woster (Medical University of South Carolina, Charleston, SC, USA) and ^2^ Dr. Tuomo Keinanen (University of Eastern Finland, Finland). ^3^ The non-toxic concentrations were selected from the preliminary experiments using the MTT assay.

**Table 2:** Mouse immunization regimens with the recombinant NS5B protein.

Experiment Series	Groups (Compounds)	Recombinant NS5B, 4 µg/Mouse	Compound Delivery Method	Dose of Compounds
1	Saline	IP ^1^ in a mixture with compounds	IP	No
	NAC		IP	4 mg/mouse
	DFMO		IP	4 mg/mouse
	Control	No	No	No
2	Saline	SC ^2^	No	No
	NAC	SC	40-mM solution in drinking water	20 mg/mouse/day (1 g/kg body weight /day)
	DFMO	SC	0.2% solution in drinking water	6 mg/mouse/day (0.3 g/kg body weight/day)
	CpG	SC in a mixture with CpG	SC	50 µg/mouse
	Control	No	No	No

Note: ^1^ IP—intraperitoneal injection; ^2^ SC—subcutaneous injection.

**Table 3:** Sequences of primers used for mRNA quantification.

Gene	Primer Sequence (5′–3′)	
	Direct	Reverse
*ODC* (ornithine decarboxylase)	CCTTGTGAGGAGCTGGTGATA	GGTCCAGAATGTCCTTAGCAGT
*Arg1* (arginase 1)	GAAGACTAGAGCCATGCGCC	TTTGAGAAAGGCGCTCCGAT
*Arg2* (arginase 2)	TCTCCTCCACGGGCAAATTC	GCAAGCCAGCTTCTCGAATG
*iNOS* (inducible NO synthase)	CTATGGCCGCTTTGATGTGC	TTGGGATGCTCCATGGTCAC
*eNOS* (endothelial NO synthase)	ATTGGCATGAGGGACCTGTG	GGGATGAGGTTGTCCTGGTG
*IFN*-*α* (interferon alpha)	CTACTGGCCAACCTGCTCTC	CTGCGGGAATCCAAAGTCCT
*IFN*-*γ* (interferon gamma)	AGCAAGGCGAAAAAGGATGC	TCATTGAATGCTTGGCGCTG
*TGF*-*β1* (transforming growth factor beta 1)	AGCTGCGCTTGCAGAGATTA	GTATCAGTGGGGGTCAGCAG
*IL*-*10* (interleukin 10)	CAGAGAAGCATGGCCCAGAA	GCTCCACTGCCTTGCTCTTA
*IL*-*12a* (interleukin 12a)	CCGAAACCTGCTGAAGACCA	GGTTTGGTCCCGTGTGATGT
*IDO1* (indolamine-2,3-dioxygenase 1)	TGGTGGAAATCGCAGCTTCT	TGCAGTGCCTTTTCCAATGC
*IDO2* (indoleamine-2,3-dioxygenase 2)	TGCCATGCTGAGCTTCTTGA	ACTGCTAAGCACCAGGACAC
*ICAM*-*1* (intercellular adhesion molecule 1)	CAGCTGCTGCTGCTTTTGAA	AGGCTACAAGTGTGCATCCC
*IRG1* (immune-responsive gene 1)	TGAGCCAGTTACCCTCCAGA	TCATCCTCTTGCTCCTCCGA
*GUS* (glucuronidase-β)	CCAGAGCGAGTATGGAGCAG	CAGATGAGCTCTCCGACCAC

## Data Availability

Not applicable.

## Supplementary Materials

The following are available online at https://www.mdpi.com/article/10.3390/ijms22136892/s1, Table S1: Immune response of the mice to HCV NS5B when administered in a mixture with compounds, Table S2: Relative content of T lymphocytes and dendritic cells in splenocytes of the mice immunized with the recombinant HCV NS5B protein.
