# Supplementary material for: Difluoromethylornithine (DFMO), an Inhibitor of Polyamine Biosynthesis, and Antioxidant N-Acetylcysteine Potentiate Immune Response in Mice to the Recombinant Hepatitis C Virus NS5B Protein
Source: Int J Mol Sci. 2021 Jun 26;22(13):6892. doi: 10.3390/ijms22136892 (PMC8268280; doi:10.3390/ijms22136892)
Supplement: Supplementary file 1 [file ijms-22-06892-s001.zip › ijms-1238700-supplementary.pdf]

International Journal of *Molecular Sciences*

MDPI

Article

Difluoromethylornithine (DFMO), an inhibitor of polyamine biosynthesis, and an antioxidant N-acetylcysteine potentiate immune response in mice to the recombinant hepatitis C virus NS5B protein

Ekaterina I. Lesnova [1], Olga V. Masalova [1,*], Kristina Yu. Permyakova [1,3], Vyacheslav V. Kozlov [1], Tatyana N. Nikolaeva [1], Alexander V. Pronin [1], Vladimir T. Valuev-Elliston [2], Alexander V. Ivanov [2,*] and Alla A. Kushch [1]

[1] Gamaleya National Research Center of Epidemiology and Microbiology, Ministry of Health of the Russian Federation, Moscow 123098, Russia; wolf252006@yandex.ru (E.I.L.); ol.mas@mail.ru (O.V.M.); kristusha164@mail.ru (K.Y.P.); hyperslava@yandex.ru (V.V.K.); tatyananik.55@mail.ru (T.N.N.); proninalexander@yandex.ru (A.V.P.); vitallku@mail.ru (A.A.K.)

[2] Center for Precision Genome Editing and Genetic Technologies for Biomedicine, Engelhardt Institute of Molecular Biology, Russian Academy of Sciences, Moscow 119991, Russia; aivanov@yandex.ru (A.V.I.); gansfaust@mail.ru (V.T.V.-E.)

[3] Federal State Budgetary Educational Institution of Higher Education "Moscow State Academy of Veterinary Medicine and Biotechnology - MVA by K.I. Skryabin", Moscow 109472, Russia; kristusha164@mail.ru (K.Y.P.)

* Correspondence: ol.mas@mail.ru (O.V.M.); aivanov@yandex.ru (A.V.I.); Tel.: +7-499-190-30-49 (O.V.M.); +7-199-135-60-65 (A.V.I.)

Table S1. The immune response of mice to HCV NS5B when administered in a mixture with compounds.

Group	Anti-NS5B antibody titers		Lymphocyte proliferation,	IFN-γ secretion,
	**IgG1**	IgG2a	SI	pg/mL
**saline**	**700±280 [1]**	35±28	1.8±0.2 [2]	430±225 [2]
NAC	790±490	54±35	1.9±0.2	471±173
DFMO	857±420	49±29	1.7±0.3	527±164
Control	15±8	11±13	1.4±0.9	20±15

Each group (5 mice) received the recombinant protein HCV NS5B at a dose of 4 μg/mice during three injections with two-week interval. NS5B was injected intraperitoneally in saline or in combination with NAC and DFMO at the dose 4 mg/mice. Humoral and cellular immunity was examined on days 9 after the third immunization. [1] Values show the geometric mean titer ± SD or [2] mean ± SD of fore measurements done from one experiment

Table S2. Relative content of T lymphocytes and dendritic cells in the splenocytes of mice immunized with recombinant HCV NS5B protein.

Group	Th CD4+	CTL CD8+	DC CD11c+
saline	11.5±1.7	3.8±0.3	3.5±0.3
NAC	12.4±0.6	4.1±0.2	4.2±0.1
DFMO	10.5±1.6	3.7±0.2	3.9±0.3
CpG	11.9±0.4	4.4±0.5	4.2±0.3
Control	12.1±1.7	3.9±0.4	3.9±0.5

Each group (5 mice) received the recombinant protein HCV NS5B at a dose of 4 μg/mice during three injections with two-week interval. NS5B was injected subcutaneously in combination with saline or CpG, whereas NAC and DFMO – with drinking water. Splenocytes from the immunized mice were stained with anti-CD4, anti-CD8, and anti-CD11c antibodies and analyzed by flow cytometry. Values are means ± SD (in the percentages) of three independent analyses, and each of them was performed in triplicate.
